# Uncovering the role of elementary processes in network evolution

**DOI:** 10.1038/srep02920

**Published:** 2013-10-10

**Authors:** Gourab Ghoshal, Liping Chi, Albert-László Barabási

**Affiliations:** 1 Department of Physics, Biology and Computer Science, Center for Complex Network Research, Northeastern University, Boston, MA 02115, USA; 2 Department of Medicine, Harvard Medical School and Center for Cancer Systems Biology, Dana-Farber Cancer Institute, Boston, MA 02115, USA; 3 Complexity Science Research Center, Institute of Particle Physics, Central China Normal University, Wuhan 430079, China; 4 Current address: Department of Earth and Planetary Sciences, Harvard University, Cambridge, MA 02138, USA.

## Abstract

The growth and evolution of networks has elicited considerable interest from the scientific community and a number of mechanistic models have been proposed to explain their observed degree distributions. Various microscopic processes have been incorporated in these models, among them, node and edge addition, vertex fitness and the deletion of nodes and edges. The existing models, however, focus on specific combinations of these processes and parameterize them in a way that makes it difficult to elucidate the role of the individual elementary mechanisms. We therefore formulated and solved a model that incorporates the minimal processes governing network evolution. Some contribute to growth such as the formation of connections between existing pair of vertices, while others capture deletion; the removal of a node with its corresponding edges, or the removal of an edge between a pair of vertices. We distinguish between these elementary mechanisms, identifying their specific role on network evolution.

The study of networks has received significant attention from the scientific community, thanks to its utility as a useful representation of many complex systems found in the real world, ranging from social to technological, infrastructural, biological and epidemiological systems^1^,^2^,^3^,^4^,^5^,^6^. While seemingly disparate, these networks show common features, among them the fact that they evolve and grow, and many display heterogeneous degree distributions^7^,^8^. A series of models have been proposed to account for the growing nature of networks and to uncover the role of various processes that affect the network topology. Perhaps the best-known are the class of models based on preferential attachment^9^, in which vertices are added to a network with edges that attach to pre-existing vertices with probabilities depending on their degrees. When the attachment probability is precisely linear in the degree of the target vertex the resulting degree distribution follows the power-law *p_k_* ~ *k*^−*γ*^. This case is of special interest because many networks from citation networks to the World Wide Web are observed to have degree distributions that approximately follow power laws^10^.

While the preferential attachment model captures the qualitative features of network evolution, it is a minimal model with obvious limitations: (i) It predicts the value of the degree exponent to be *γ* = 3, whereas most real world networks have exponents in the range 2 ≤ *γ* ≤ 4. (ii) It predicts a pure power law degree distribution, while real systems are characterized by small degree saturation and high-degree cutoffs. (iii) It ignores a number of elementary processes that play an important role in the evolution of many real networks, like the addition of internal links and node or link removal.

To account for these limitations, a considerable amount of research has been conducted in the network science community, exploring a series of pertinent modifications to the original model, by changing the form of the attachment probability^11^,^12^,^13^,^14^,^15^,^16^, incorporating effects such as ageing^17^,^18^,^19^,^20^, fitness^21^,^22^,^23^, and allowing for the simultaneous addition and deletion of edges and vertices^24^,^25^,^26^,^27^,^28^,^29^, each leading to predictions that approximate better the degree distributions observed in real systems. Despite these advances, the current models were motivated by specific problems, making it difficult to understand the role of *individual* processes on network evolution. For example, models have typically included both random and preferential *external* attachment of nodes^12^,^15^, or preferential external and *internal* addition of nodes and edges^13^,^16^, but not *simultaneously* incorporating all of these, nor in a fashion that the individual role of each process can be separately elucidated. At the same time these models neglected the important role of the *deletion* of nodes and edges. When considered^24^,^25^,^26^,^27^,^28^,^30^, this was studied in conjunction only with preferential attachment of new nodes, and although the qualitative results were sound (namely that deletion increases the value of *γ*, eventually driving the network from a power law to an exponential regime) the predictions for the degree exponent *γ* > 3 even in the presence of low deletion rates, was not in agreement with what is seen in real networks. Furthermore, when attempts were made to incorporate simultaneously multiple growth processes, the models were parameterized such that it is difficult to separate the contributions of the individual elementary processes to the network topology. For example, Ref. 31 considered the combination of adding links between existing nodes and random rewiring of edges along with node addition. Unfortunately the variable representing each process were dependent parameters, making it difficult to “decouple” their role on the evolution from each other.

In light of these difficulties, our goal here is to study in detail a model, which contains the fundamental processes by which a network evolves, along with the degree of freedom of being able to study and emphasize the role of each process *independent* of the other. Our primary goal is not necessarily to uncover new results (although we do present a series of new findings) but rather separate the “wheat from the chaff” untangling the results of previous work, thus putting in context and interpreting the role of the individual growth processes.

To be specific, in this paper we study a model which incorporates some of the most *elementary* processes that drive network evolution, namely the addition of vertices and edges and their removal. Broadly speaking there are four distinct microscopic mechanisms that contribute to network evolution. Two contribute to growth, i.e. either a new vertex attaches to pre-existing vertices, or an existing pair of vertices form connections between them. The other two capture deletion, either the removal of an existing node with its corresponding edges, or the removal of an existing edge between a pair of vertices. We systematically distinguish between these four fundamental processes, identifying their role on network evolution and the degree distribution. We show that one can generate networks with degree distributions in the same range as measured in real networks, in the presence of either specific combinations of these processes, or indeed all of them occurring simultaneously.

## Results

### Model for network evolution

Let *p_k_* denote the fraction of vertices that have degree *k* in a network of size *n*. Following^11^,^12^ we define the attachment kernel *π_k_* to be *n* times the probability that a given edge of a newly added vertex attaches to a pre-existing vertex of degree *k*. The factor *n* here is convenient, as it means that the total probability that the given edge attaches to any vertex of degree *k* is *π_k_p_k_*. Since each edge must attach to a vertex of some degree, *π_k_* must satisfy the normalization condition, 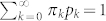. We define *π_k_*_,*k*′_ to be the joint attachment kernel for an edge to be placed between the two vertices of degree *k* and *k*′. The correct normalization in this case is given by 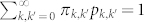, where *p_k_*_,*k*′_ is the joint degree distribution. We consider a network that evolves in time, according to the four basic processes outlined in the introduction. That is, in each unit of time, the following elementary steps are considered:

#### Node addition

We add a new vertex to the graph along with *c* edges. While in principle, the number of these edges can be drawn from some distribution, in the spirit of simplicity, we assume that all newly added vertices have the same degree. We must next decide how to attach the *c* edges to pre-existing vertices in the network via the attachment kernel *π_k_*. In the preferential attachment model *π_k_* ~ *k* (ref. 9), which precludes nodes with initially no links (*k* = 0) from acquiring an edge. In real networks however even isolated nodes can acquire links. Indeed, in citation networks, a new research paper has a finite probability of being cited, or in social networks, a person that moves to a new city will quickly acquire acquaintances. Zero-degree nodes can acquire links if we add a constant *a* to *π_k_*^15^,^32^, obtaining 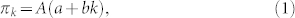
where *A* = (*a* + *b*〈*k*〉)^−1^. Note that for *k* = 0, *π_k_* ~ *a*, thus this represents the probability for a node to acquire its first link and can be thought of as its initial attractiveness^23^ or *fitness*^21^,^22^. In the limit *a* → 0 we recover pure preferential attachment, while as *b* → 0, *π_k_* = 1 and we have purely random attachment of vertices leading to an exponential degree distribution^33^.

#### Addition of internal links

Often links do not arrive with new nodes, but are added between those already extant in the network. For example, the vast majority of the links in the World Wide Web are internal links, corresponding to URL's added between existing web documents, and so are virtually all new social/friendship links formed between individuals who already have other friends. To reflect this, we select *m* pairs of vertices already present in the graph and according to *π_k_*_,*k*′_ add a single edge between them. In order to choose the form of *π_k_*_,*k*′_, we take inspiration from measurements made on real networks^34^,^35^, suggesting that internal links are formed with probability *π_k_*_,*k*′_ ~ (*s* + *tk*)(*s*′ + *t*′*k*′), incorporating both random (*s*, *s*′) and preferential (*t*, *t*′) attachment. This form allows us to factorize the joint probability *π_k_*_,*k*′_ into the product 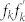, and assuming *s* = *s*′, *t* = *t*′, we choose, 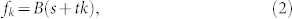
where *B* = (*s* + *t*〈*k*〉)^−1^.

#### Node deletion

Many real systems also experience node *deletion*, reflecting for example, the departure of an employee from an organization or the removal of a document from the WWW. To account for this phenomenon, with probability *r* we randomly remove a single vertex from the graph, such that *r* < 1 corresponds to a growing network, while *r* = 1 represents a network of fixed size where deletion is balanced by growth. In principle *r* can be greater than 1 (of course then it ceases to be a probability), in which case we have *shrinking* networks^36^,^37^,^38^ that eventually disintegrate. In this paper, however, we restrict ourselves to the case of growing networks and thus exclude this case.

#### Link deletion

Finally networks may also experience the deletion of individual links between nodes. In fact this is probably more common than node deletion, as URL's between webpages are frequently removed or relationships between friends in a social network are terminated while they continue to maintain ties with other acquaintances. Therefore with probability *q* we randomly select *m* existing pairs of vertices and remove the edge between them.

**Figure 1:**
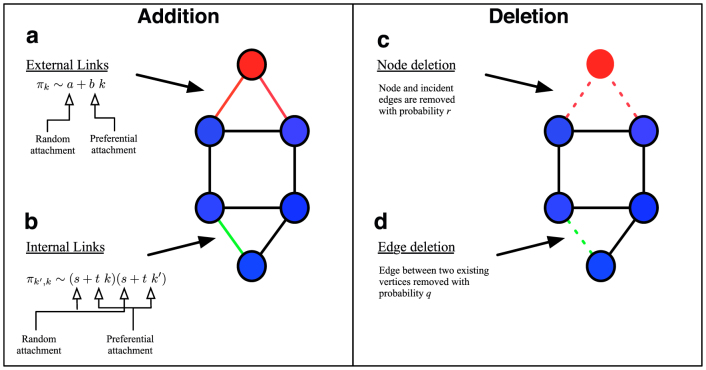
Summary of the elementary processes whose input on network topology is studied in the paper. *Left panel* (a) With probability *π_k_* ~ *a* + *bk* a newly introduced vertex (red circle) connects to *c* existing vertices (in this case *c* = 2). (b) With probability *π_k_*_′,*k*_ a pair of existing vertices form a connection to each other (green line). *Right panel* (c) With probability *r* a vertex (red circle) is chosen at random and deleted with its edges (dashed red lines). (d) With probability *q* an edge between two existing vertices (green dashed line) is removed.

We thus have eight parameters in the model, their role being summarized in Table 1, while the four processes captured by the model are schematically illustrated in Fig. 1. Now that we have our basic ingredients, we can write down a rate equation that captures the evolution of the resulting network, 
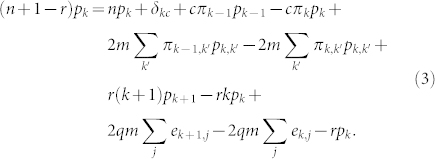
The term *δ_kc_* represents the addition of a vertex with degree *c*, while *π_k_* represents the flow of degrees from from *k* − 1 to *k* and *k* to *k* + 1 owing to the addition of a single edge from the new vertex. Terms involving *π_k_*_,*k*′_ represent the flow of degrees due to the addition of a single edge between two existing vertices, while the combinatorial factor 2 accounts for the fact that each end of the edge can connect to a vertex with degree *k* or *k*′. The terms (*k* + 1)*p_k_*_+1_ and *kp_k_* describe the flow from degree *k* + 1 to *k* and from *k* to *k* − 1 as vertices lose edges when one of their neighbors is removed from the network. The term *rp_k_* represents removal of a vertex of degree *k* with probability *r*, while *e_k,j_* is the probability that a randomly selected edge has a vertex of degree *k* on one end and another of degree *j* on the other. Contributions from processes in which a vertex gains or loses two or more edges in a single unit of time vanish in the limit of large *n* and have been neglected.

The rate equation (3) is fairly complex due to the presence of the joint probabilities *π_k_*_,*k*′_ and *p_k_*_,*k*′_. However if we assume that the network lacks degree correlations, then *p_k_*_,*k*′_ can be factorized as *p_k_p_k′_*, while Σ*_j_e_k_*_,*j*_ = *kp_k_*/〈*k*〉. With the aid of generating functions (Supplementary Methods, Sec. S1) this can be recast in differential equation form thus, 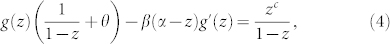
where *g*(*z*) = Σ*_k_* *p_k_z^k^* and *θ*, *β*, *α* are functions of the parameters listed in Table 1. (See Supplementary Methods, Eq. (S3) for their explicit forms.)

### Average degree 〈*k*〉

The solution to Eq. (4) is non-trivial. We can make progress, however, if the average degree 〈*k*〉 depends only on the free parameters of the model *c*, *m*, *r*, *q*. Note that at each time step the net number of vertices added is 1 − *r*, each of which has *c* edges. There are *m* edges added between existing pairs of vertices, while the average number of edges removed when removing a randomly chosen vertex is by definition 〈*k*〉. The number of edges removed between existing pairs of vertices is *q* × *m*. Therefore in each time-step the mean number of edges added is *c* + *m* − *r*〈*k*〉 − *qm*. For a graph with *e* edges and *n* vertices 〈*k*〉 = 2*e*/*n*. After time *τ* we have *n* = (1 − *r*)*τ* and assuming that 〈*k*〉 has an asymptotically constant value, *e* = (*c* + *m* − *r*〈*k*〉 − *qm*)*τ*. Substituting and re-arranging, we obtain 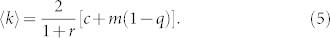


### Solutions for the degree distribution *p_k_*

We proceed to solve Eq. (4) to determine the degree distribution *p_k_*. While we can solve the equation with all its components numerically, it is difficult to get a closed form analytic expression. We therefore first treat the case with only the node and link addition processes (which we can solve exactly) and then use an approximation to include the deletion processes.

#### Pure growth

We start by considering the case when vertices and edges are added but never removed (left panel of Fig. 1). In this case *r*, *q* = 0 and thus *α* = 0. With this simplification, and after a sequence of manipulations (Supplementary Methods, Sec. S2) it can be shown that this leads to a degree-distribution, 
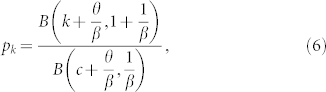
where *B*(*x*, *y*) = Γ(*x*)Γ(*y*)/Γ(*x* + *y*) is the Beta function. For large *x*, we have *B*(*x*, *y*) ≈ *x^−y^* and thus asymptotically *p_k_* ~ (*k* + *k*_0_)^−*γ*^, a shifted power-law, where, 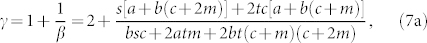

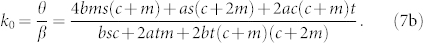


**Figure 2:**
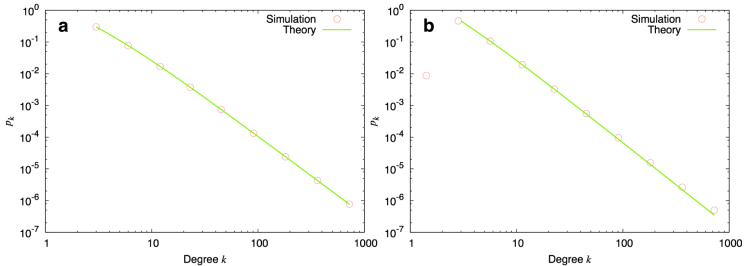
Comparison between numerical simulations and analytical calculations (a) The points represent the result of logarithmically binning numerical simulations over multiple realizations of the evolution process of a growing network involving pure addition with parameters *c* = 3, *m* = 2, *a* = 0.5, *b* = 1, *s* = 0.5, *t* = 1.0. The final size of the network is *n* = 10^5^ nodes. The solid line is the theoretical result Eq. (6). The agreement between the two is excellent. (b) Same set of parameters, now including deletion processes with *r* = 0.2, *q* = 0.1. The solid line is a fit to the form *p_k_* ~ (*k* + *k*_0_)^−*γ*^ where *γ* is the theoretical expression (10). Once again the agreement is very good.

In Fig. 2a we plot *p_k_* as a result of numerical simulations of the evolution process described here, along with the theoretical expression (6). As the figure shows the agreement between the two is excellent.

We can isolate the effect of each growth process by setting its associated parameter to 0. The combinations are listed in Table 2, which allows us to draw the following conclusions.

### Initial attractiveness/random attachment

Once present these two have the following consequences:

- *Increases the degree exponent γ*. As we see from Table 2 its primary effect is to introduce positive contributions to the exponent *γ*, making the network more *homogenous*. For example in the simple case of preferential and random attachment of external links, we have, 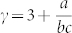. This means that *γ* is always greater than 3 and therefore the second moment 〈*k*^2^〉 is finite affecting both network robustness^39^,^40^,^41^,^42^ and spreading phenomena^43^,^44^. In general the contributions are simple additive perturbations for each random process, in combination with internal *or* external preferential attachment. When both external and internal preferential attachment are present then the perturbations are more complex combinations than simple linear additive terms, however the qualitative behavior is the same, *γ* increases.
- *Generates a small-degree cutoff*. We see that the solution is a shifted power law *p_k_* ~ (*k* + *k*_0_)^−*γ*^, implying a small-degree saturation at *k*_0_, where *k*_0_ is a function of the parameters *c*, *m*, *a*, *b*, *s*, *t*. In the limit 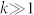, however this initial attractiveness loses relevance and *p_k_* has a purely power law tail, a phenomenon that can be understood from the fact that initial attractiveness predominantly favors small-degree nodes.

### Internal links

To understand the role of internal links we consider several special cases.

- *Double random attachment* (*t* = 0, *a*, *b*, *s* ≠ 0*)*. In this case we have external preferential and random attachment as well as random addition of internal links. The degree exponent resulting from this evolution process is 
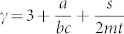
. Therefore–like in the case of external links–random attachment of internal links continue to play a homogenizing role as the exponent *γ* > 3 for any combination of the parameters *m*, *s*, *t*. Indeed the random addition of internal links tends to favor lower degree nodes due to their preponderance, and consequently make them more similar to hubs by increasing their degree. In the limit where random–dominates over preferential–attachment (*a*, *s* → ∞) the distribution converges to the exponential universality class as 〈*k*^2^〉 is finite.
- *Double preferential attachment* (*a*, *s* = 0 & *b*, *t* ≠ 0). In the case of pure double preferential attachment, both ends of a new link are proportional to the degrees *k*, *k*′ of the nodes they connect. The resulting exponent is 
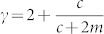
, indicating that it varies between 2 and 3. Thus in this case, we see that the preferential addition of internal links makes the network more *heterogenous*. This is the result of two effects. Preferential attachment of external links creates a power law network with hubs (albeit with a fixed *γ* = 3), whereas the internal links preferentially connect high-degree nodes allowing them to grow faster at the expense of low-degree nodes, lowering *γ* below 3.
- *Random and Preferential attachment*. In this case all parameters are non-zero, and the overall effect is a combination of the two listed above. The key thing to note, is that the range of the degree exponent is 2 < *γ* < ∞.

The most important phenomenon that we glean from the results is the heterogenizing influence on *p_k_* when internal links are added preferentially. Even in combination with the other effects, there are parameter ranges where *γ* < 3 and since most real networks are known to have exponents in this range, this suggests that internal preferential attachment plays a key role in maintaining the documented heterogeneity in real networks. Next we examine whether node and edge deletion preserves or destroys the effects of the elementary growth processes.

#### Growth with deletion

In the presence of node and edge deletion, solving Eq. (4) in closed form is difficult. However, as we are primarily interested in the asymptotic form of the degree distribution, we resort to approximation methods to determine the form of *p_k_* in the tail of the distribution. In order to do so, we first simplify the expression for the attachment kernels by setting *b*, *t* = 1, such that *π_k_* = *A*(*a* + *k*) and *f_k_* = *B*(*t* + *k*). The disadvantage of this is that we cannot treat random and preferential attachment separately. However we have already explored the homogenizing role of random processes in the previous section and based on the limiting behaviors that we found, we assume *p_k_* follows a power-law with an exponential correction, *p_k_* = *Ck^−γ^*Ω*^k^*, and solve for *γ* and Ω in the limit 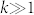. Next, following^14^,^45^, we employ the method of telescoping products via an expansion of *p_k_* at large *k* (Supplementary Methods Sec. S2). Substituting this expansion into Eq. (3) we find two solutions for Ω, namely Ω = 1 and 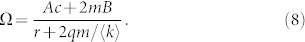
If Ω < 1 then the solution (8) is normalizable and *p_k_* decays exponentially (with a power-law correction). However if the ratio is greater than 1, it does not correspond to a normalizable probability distribution and therefore the correct solution is Ω = 1, leading to a purely power law distribution *p_k_* ~ *k^−γ^*. This suggests that one of the primary effects of the deletion process is to induce a *topological phase transition* at the point Ω = 1, separating an exponential regime from a power-law regime. This phase transition has previously been pointed out by^45^, in the limited context of node addition and deletion. We find that this phenomenon is robust to the inclusion of the full set of growth processes considered here.

With a further simplifying assumption, we can define a single critical parameter that determines the scaling regime. If *a* = *s*, or in other words the degree of external and internal random attachment is the same, then (8) reduces to: Ω = *A*(*c* + 2*m*)/(*r* + 2*qm*/〈*k*〉). Substituting in the expressions for *A*, 〈*k*〉, we find 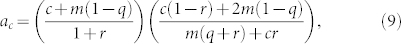
a strictly positive quantity, such that for *a* > *a_c_* the distribution is exponential, whereas for *a* < *a_c_* the distribution follows a power-law. At the critical point *a_c_* it can be shown that *p_k_* has the stretched exponential form 
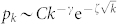
 (Supplementary Methods, Sec. S2).

Below *a_c_* we find 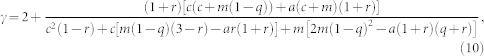
and in Table 3 we list the exponent *γ* as a function of different parameter combinations, now including deletion. In Fig. 2b, we plot *p_k_* as a result of numerical simulations of the evolution process and compare it to the theoretical expression (10), finding that the agreement between the two is very good.

We can define one more critical point within the power-law regime, in terms of a critical parameter 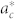 that separates power-laws with finite second moments (i.e *γ* > 3) and those with infinite second moment (2 < *γ* ≤ 3). To do so we set (10) equal to 3 and solve for *a* to find 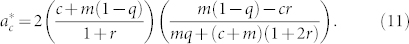
Therefore for 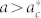 we have a power-law with exponent *γ* > 3 and for 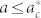 we have 2 < *γ* ≤ 3. Note that negative values of 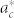 are possible. In fact certain authors have suggested^12^ that one can generate power-laws with exponent *γ* < 3 if the parameter *a* is negative (one can see this in Table 2 by setting either *a*, *b* < 0). It is however unclear as to what a negative value of *a* might mean. The most logical way to interpret *a* is either as random attachment, or a fitness/intial attractiveness parameter, and there does not seem to be a reasonable argument for a vertex to have negative fitness. Consequently, we require *a* > 0, allowing us to define yet another critical value, 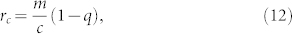
such that if *r* > *r_c_* the phase with 2 < *γ* ≤ 3 disappears (as 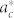 is negative) leaving us only with an exponential phase and a power-law phase with *γ* > 3. This is fairly easy to understand if the condition is recast in a different form. Recall that the *existence* of the state (2 < *γ* ≤ 3) is driven by internal preferential attachment which is parameterized by *m*. Using (5) we can rewrite Eq. (12) as the condition *r*〈*k*〉 + 2*mq* > 2*m*. The term *r*〈*k*〉 + 2*mq* however is just the average number of links that are removed in a given time-step through node and edge deletion, whereas 2*m* is the number of internal links added via preferential attachment. So if the number of deleted links is greater than the number of internal links added, than the effect of internal preferential attachment is suppressed and therefore the state vanishes.

**Figure 3:**
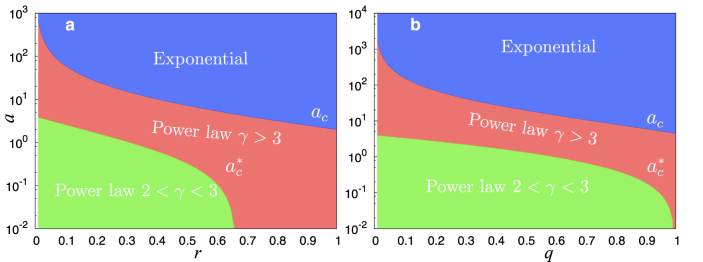
The existence regions of the three phases as determined by the elementary mechanisms (a) The random attachment parameter *a* as a function of *r* (node removal) with *q* (edge-removal) set to zero and *c* = 3, *m* = 2. The three phases are separated by the boundaries *a_c_* (9) and 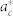 (11). Above *a_c_* the degree distribution is exponential (blue). At the phase boundary *a_c_* the distribution is a stretched exponential of the form 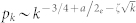. Below *a_c_* the distribution is a power-law *p_k_* ~ *k*^−*γ*^ (red), while below 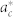 the distribution is a power-law with infinite second moment, i.e 2 < *γ* < 3 (green). (b) Same as (a) but now as a function of *q*, with *r* set to 0. We find access to a much larger region of phase-space permitting the power-law regime, specially the green phase.

In Fig. 3 we plot the three phases, exponential (blue), power-law *γ* > 3 (red) and power-law 2 < *γ* ≤ 3 (green), also showing the random attachment parameter *a* as a function of the deletion parameters *r*, *q*. In order to separate the effects of node and edge deletion we set *q* = 0 in Fig. 3a and *r* = 0 in 3b. In both cases, we have the existence of three phases separated by the *a_c_*, 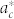 curves. However, we see that edge deletion permits a much larger range in the phase-space for the existence of a power-law degree distribution (especially for the green phase). The critical parameters *a_c_*(*c*, *m*, *r*, *q*) and 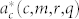 in conjunction with Table 3 allow us to discuss the effects of each deletion process.

### Node deletion

Node deletion has a strong homogenizing effect on the degree distribution, inducing a topological phase transition from a power law to an exponential phase. Its effect is better understood by looking at specific limits.

- (*a*, *q* = 0) When including only external preferential attachment, we have 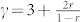. For *r* < 1, the number of removed nodes is less than that of newly introduced nodes and hence the network exhibits net growth. However *γ* increases in function of *r* and thus the network is more homogenous. Specifically, in the limit *r* → 1, we see that *γ* diverges when there is only external preferential attachment, and the degree distribution transitions to a stretched exponential^27^. This can be explained by the fact that random node removal serves as a pruning of the degree of the high-degree nodes (since the nodes that are being removed are more numerous low degree ones which are connected to the hubs resulting in a peak for *p_k_* near 〈*k*〉). Thus when the addition of a node is compensated by the deletion of one, the increase of neighbors of hubs (from the addition of the new node) is balanced by the removal of its low degree neighbors, ultimately resulting in the homogenization of the network.

On the other hand in the presence of internal preferential attachment the degree exponent is 
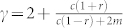
 and one can see that the divergence is suppressed. The adverse effect of deletion is compensated by preferentially connecting hubs together, thus maintaing the heterogenous character of the network. In this regime all three phases can co-exist, although at *r* = *r_c_* the green state vanishes for reasons explained earlier.

- (*a* ≠ 0, *q* = 0). The compensating effect of internal preferential attachment is eventually overcome with the introduction of random addition. The homogenizing effect of this, in conjunction with node deletion eventually induces a topological phase transition between a power law (red) and exponential (blue) phases at *a* = *a_c_*, which in this parameter regime is 
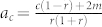
. Note, that *a_c_* ≥ 0 for all *r*, and therefore the phase transition exists whenever there is *any* node deletion. In addition to this the power law region is separated into the red and green phases at 
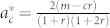
. One again, the green state vanishes at *r* = *r_c_*.

### Edge deletion

Edge deletion has a similar effect to node deletion, however, it admits a wider region in phase space for power laws. We once again examine these by looking at different limits.

- (*a*, *r* = 0) The value of the exponent in this regime is 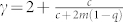. Unlike node deletion, we see that *γ* ranges between 2 and 3 for all values of *q*. In the limit *q* → 1, *γ* = 3. This is easy to understand, since for *q* = 1 the number of internal edges added and those removed are the *same*, and thus we effectively only have preferential attachment of external edges and recover that limit.
- (*a* ≠ 0, *r* = 0) In this limit, just as in node deletion, there is a phase transition at 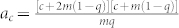. Once again, we see that the phase transition is present for all values of *q*. The curve marking the separation between the green and red phases is now, 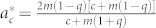, which is positive for any *q*. So unlike the case for node deletion, the green phase exists for all values of *q*.

### Node and edge deletion

In general all three phases co-exist. The corresponding limiting behaviors are:

- *Homogenous regime* (*r* = *r_c_*) The green phase vanishes and only two phases survive (blue and red).
- *Exponential regime* (*r*, *q* → 1) Red and green phases vanish and only the blue phase survives.
- *Heterogenous regime* (*r*, *q* → 0) The blue phase vanishes while the green and red phases survive.

## Discussion

Taken together the results suggests that the form of the degree distribution *p_k_* is in general a highly complex interplay between the different parameters, and is determined by the dominant elementary process. While the combined effect is complex, we have been able to clearly outline the role of the individual mechanisms, which are:

### External links

A pure power law with *γ* = 3 emerges if the links are added only via preferential attachment. This can be thought of as the “backbone” or starting point for understanding the degree distribution of real networks. When one includes initial attractiveness of nodes or random attachment of the links, these lead to a small degree saturation of the distribution by introducing a shift *k*_0_. Furthermore it homogenizes the network by making *γ* > 3, driving it toward the exponential universality class.

### Internal links

When placed between nodes randomly, internal links have the same effect as initial attractiveness. However, when preferentially added, they tend to link together high degree nodes, allowing them to grow faster than low degree ones and thus the resulting network becomes more heterogenous. In conjunction with external preferential attachment this lowers the exponent to *γ* < 3.

### Node and edge deletion

Taken together node and edge removal have a disruptive influence on the network topology. Random node removal depletes the low-degree nodes (since they are more numerous) while random edge removal depletes the high-degree nodes (since they have the most links) and their combined effect is to drive the exponent *γ* far from 3, thus making the network more homogenous. In particular as *r* → 1 the power law form of the distribution is destroyed and the network undergoes a topological phase transition to a stretched exponential. When combined with random attachment (parametrized by *a*), this happens for *r*, *q* < 1 at a critical value *a_c_* such that for *a* > *a_c_* the network has an exponential distribution whereas for *a* < *a_c_* it continues to follow a power-law. The power-law phase includes a region with 2 < *γ* ≤ 3 (due to internal link addition). This however vanishes for *r* > *r_c_*, when the number of deleted links exceeds the number of added *internal* links.

Thus random attachment and deletion act as homogenizing forces, conspiring *against* the heterogenizing force, preferential attachment. The resulting degree distribution, whether exponential or power law, depends on which of these dominate. However, the important thing to note is that there are wide regions in phase space of Fig. 3 that permit networks with *γ* < 3 when *all* of these elements co-exist. This is particularly important as in most real networks (for which we have 2 < *γ* ≤ 4), several of the elementary processes discussed here *do* appear together. In citation networks, for example, there are no deletion effects (in principle citations can be retracted, but this is rare) although empirical measurements suggest the presence of initial attractiveness and preferential attachment^46^. Our results indicate that the degree exponent should be a shifted power law with *γ* > 3 and this is precisely what is found^47^. Many other networks, where deletion effects are present, have degree exponents *γ* < 3 and our findings indicate that the reason for observed forms for *p_k_* strongly depends on the presence of preferential attachment of internal links (in combination with external links), as well as net growth, where vertex and edge addition *outstrip* their deletion.

One can augment the findings here by generalizing the framework to directed networks^15^,^48^, including non-linear corrections to preferential attachment^12^,^14^, increasing average degree^49^,^50^,^51^, edge rewiring^22^ or aging of vertices^17^,^18^,^19^,^20^ among other effects. Each of these will of course introduce perturbations to our solutions, but the qualitative behavior should remain within the bounds determined by the *elementary* mechanisms discussed here.

## Methods

### Solving for *p_k_* using generating functions

Given a rate equation involving *p_k_* and of the form 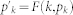, we can convert this into a differential equation by the use of generating functions 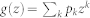. Multiplying the rate equation by *z^k^*, summing over *k* and noticing that terms in *kp_k_* can be written as *dg*(*z*)/*dz*, we arrive at a differential equation of the form *dg*(*z*)/*dz* = *F*(*g*(*z*)). Assuming that a solution to the differential equation exists in closed form (typically special functions like the Beta function or Hypergeometric functions), this can then be expanded in a power series of *z*, following which *p_k_* is determined by comparing coefficients.

### Solving for *p_k_* using telescoping products

Frequently a closed form solution to such a differential equation does not exist. Nevertheless one can make progress if one is interested in the form of the distribution for large *k* (the tail of *p_k_*). Typically a guess is made to the general form of *p_k_* either through heuristic arguments or by examining the results of numerical simulations. In the case discussed in this manuscript, we chose *p_k_* = *Ck*^−*γ*^Ω*^k^*. A high degree expansion is then performed for the telescoping products *p_k_*/*p_k_*_−1_ and *p_k_*/*p_k_*_+1_ in powers of 1/*k*, which is then substituted back into the rate equation Eq. (3). Ignoring terms in 1/*k* (since we are interested in the limit 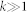) and setting terms in *k* to zero gives us solutions for Ω. Depending on the regime we are interested in, the corresponding solution for Ω is substituted back into the equation and setting the *k*-independent term to zero gives us the solution for *γ*.

## Author Contributions

G.G., L.C. and A.-L.B. designed and performed the research and wrote the manuscript.

## Supplementary Material

Supplementary Information Supplementary Methods

## Figures and Tables

**Table 1:** List of key parameters in the model and their respective roles

Parameter	Role
*c*	Number of external edges added
*m*	Number of internal edges added/removed
*r*	Probability of removing a vertex
*q*	Probability of removing an edge
*a*	Controls random external edge addition
*s*	Controls random internal edge addition
*b*	Controls preferential external edge addition
*t*	Controls preferential internal edge addition

**Table 2:** The list of solutions as a function of the different parameters for Eq. (7). The acronyms stand for Preferential (*P*) and Random (*R*), while the subscripts refer to external (*e*) and internal (*i*). When appropriate, citations to the literature where the results have been calculated for the partial case are shown

Parameters	Evolution Process	Exponent *γ*	Range	Shift *k*_0_	Refs.
*c*, *b*	*P_e_*	3	—	0	9
*c*, *b*, *a*	*P_e_* + *R_e_*	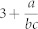	(3,∞)	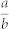	12,15
*c*, *m*, *b*, *s*	*P_e_* + *R_i_*	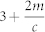	(3, 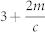 )	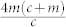	—
*c*, *m*, *b*, *a*, *s*	*P_e_* + *R_e_* + *R_i_*	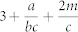	(3,∞)	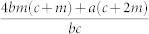	—
*c*, *m*, *b*, *t*	*P_e_* + *P_i_*	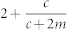	(2,3)	0	13,16
*c*, *m*, *t*, *a*	*P_i_* + *R_e_*	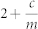	(2, 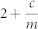 )	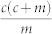	—
*c*, *m*, *t*, *a*, *s*	*P_i_* + *R_e_* + *R_i_*	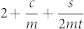	(2,∞)	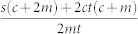	—
*c*, *m*, *b*, *t*, *a*	*P_e_* + *P_i_* + *R_e_*	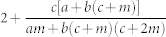	(2, 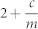 )	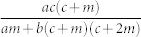	13
*c*, *m*, *b*, *t*, *s*	*P_e_* + *P_i_* + *R_i_*	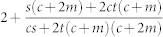	(2, 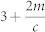 )	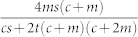	13

**Table 3:** The list of solutions of Eq (10) in function of the relevant model parameters. The acronyms stand for Preferential (P), Random (R), Deletion (D), while the subscripts refer to external (e), internal (i), node (n) and link (l). When appropriate, citations to the literature where the results for the partial case have been calculated before, are shown. Note here, that when ∞ is shown in the range column, it denotes a phase transition from a power law to an exponential regime

Parameters	Evolution process	Exponent *γ*	Range	Refs.
*c*, *r*	*P_e_* + *D_n_*	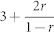	(3,∞)	24,25,26,27
*c*, *a_e_*, *r*	*P_e_* + *R_e_* + *D_n_*	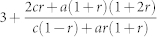	(3,∞)	45
*c*, *m*, *r*	*P_e_* + *P_i_* + *D_n_*	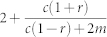	(2,∞)	—
*c*, *m*, *a_e_*, *r*	*P_e_* + *P_i_* + *R_e_* + *D_n_*	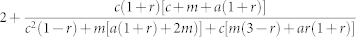	(2,∞)	—
*c*, *m*, *a_i_*, *r*	*P_e_* + *P_i_* + *R_i_* + *D_l_*	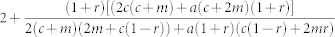	(2,∞)	—
*c*, *m*, *a_e_*_,*i*_, *r*	*P_e_* + *P_i_* + *R_e_* + *R_i_* + *D_n_*	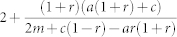	(2,∞)	—
*c*, *m*, *r*, *q*	*P_e_* + *P_i_* + *D_n_* + *D_l_*	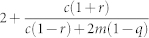	(2,∞)	—
*c*, *m*, *q*	*P_e_* + *P_i_* + *D_l_*	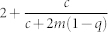	(2,3)	6
*c*, *m*, *a_e_*, *q*	*P_e_* + *P_i_* + *R_e_* + *D_l_*	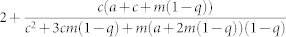	(2,∞)	—
*c*, *m*, *a_i_*, *q*	*P_e_* + *P_i_* + *R_i_* + *D_l_*	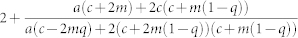	(2,∞)	—
*c*, *m*, *a_e_*_,*i*_, *q*	*P_e_* + *P_i_* + *R_e_* + *R_i_* + *D_l_*	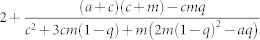	(2,∞)	—
