# Supplementary material for: Uncovering the role of elementary processes in network evolution
Source: Sci Rep. 2013 Oct 10;3:2920. doi: 10.1038/srep02920 (PMC3794373; doi:10.1038/srep02920)
Supplement: Supplementary Information — Supplementary Methods [file srep02920-s1.pdf]

Supplementary Material

Uncovering the role of elementary processes in network evolution
by Gourab Ghoshal, Liping Chi and Albert-László Barabási

Table of Contents

S1 Converting the rate equation to a differential equation **S-2**

S2 Solving for the degree distribution p_k **S-2**
S2.1 Pure growth S-3
S2.2 Growth with deletion S-3
S2.3 Stretched exponential at the critical point a_c S-4

S1 Converting the rate equation to a differential equation

The rate equation in the manuscript Eq.(3) is hard to treat analytically, primarily due to the presence of the joint probabilities $\pi_{k,k'}$ and $p_{k,k'}$. This can be simplified, however, if we make the assumption that our network lacks degree correlations (or has vanishingly small values for the correlation). In this case, $p_{k,k'}$ can be factorized as $p_k p_{k'}$, while $\sum_j e_{k,j} = kp_k/\langle k\rangle$, therefore Eq. (3) can be written as,

$$\begin{aligned} p_k \;&=\; \delta_{kc} + c\pi_{k-1}p_{k-1} - c\pi_k p_k + 2mf_{k-1}p_{k-1} - 2mf_k p_k \\ &\quad + \left(r + \frac{2qm}{\langle k\rangle}\right)(k+1)p_{k+1} - \left(r + \frac{2qm}{\langle k\rangle}\right)kp_k. \end{aligned} \tag{S1}$$

Defining the generating functions,

$$f(z) = \sum_{k=0}^{\infty} \pi_k p_k z^k, \quad g(z) = \sum_{k=0}^{\infty} p_k z^k \text{ and } h(z) = \sum_{k=0}^{\infty} f_k p_k z^k,$$

multiplying by z^k, summing over k and inserting the attachment kernels from Eqn. (1) and (2) we arrive at the differential equation,

$$g(z)\left(\frac{1}{1-z} + \theta\right) - \beta(\alpha - z)g'(z) = \frac{z^c}{1-z}, \tag{S2}$$

where,

$$\begin{aligned} \theta \;&=\; (1+r)\left(\frac{ac}{2b[c+m(1-q)] + a(1+r)} + \frac{2sm}{2t[c+m(1-q)] + s(1+r)}\right), \\ \beta \;&=\; (1+r)\left(\frac{bc}{2b[c+m(1-q)] + a(1+r)} + \frac{2tm}{2t[c+m(1-q)] + s(1+r)}\right), \\ \alpha \;&=\; \frac{cr + m(q+r)}{c + m(1-q)} \times \frac{1}{\beta}. \end{aligned} \tag{S3}$$

S2 Solving for the degree distribution p_k

The expression in Eq. (S2) can be solved via numerical methods. However, we are interested in the explicit form of p_k. Unfortunately the equation in its complete form does not lend itself well to analytical techniques. We can make progress however by considering specific cases. We start by neglecting deletion processes, and consider the case of pure growth, which can be solved exactly. Following this, we will resort to approximation methods to solve the evolution process including deletion.

S2.1 Pure growth

When vertices and edges are added but never removed, we have both $r, q = 0$ and thus $\alpha = 0$. With this modification, and using $(z/(1-z))^{1/\beta} \times z^{\theta/\beta}$ as an integrating factor, $g(z)$ is provided by

$$g(z) = \frac{1}{\beta}\left(\frac{z}{1-z}\right)^{-1/\beta} z^{-\theta/\beta} \int_0^z \frac{t^{c-1+(1/\beta)(1+\theta)}}{(1-t)^{1+1/\beta}} dt. \tag{S4}$$

Changing variables to $y = t/z$ we have,

$$g(z) = \frac{1}{\beta}(1-z)^{1/\beta} z^c \int_0^1 \frac{y^{c-1+(1/\beta)(1+\theta)}}{(1-yz)^{1+1/\beta}}. \tag{S5}$$

Expanding in powers of z and isolating the coefficients we find that,

$$p_k = \frac{B\left(k + \frac{\theta}{\beta}, 1 + \frac{1}{\beta}\right)}{B\left(c + \frac{\theta}{\beta}, \frac{1}{\beta}\right)}, \tag{S6}$$

where $B(x, y) = \Gamma(x)\Gamma(y)/\Gamma(x + y)$ is the Beta function. For large x, we have $B(x, y) \approx x^{-y}$ and thus asymptotically,

$$p_k \sim (k + k_0)^{-\gamma},$$

a shifted power-law, where,

$$\gamma = 1 + \frac{1}{\beta} = 2 + \frac{s[a + b(c + 2m)] + 2tc[a + b(c + m)]}{bsc + 2atm + 2bt(c + m)(c + 2m)}, \tag{S7a}$$

$$k_0 = \frac{\theta}{\beta} = \frac{4bms(c + m) + as(c + 2m) + 2ac(c + m)t}{bsc + 2atm + 2bt(c + m)(c + 2m)}. \tag{S7b}$$

S2.2 Growth with deletion

We have established that the pure growth process leads to a power law distribution. Previous work [27, 45] indicates that the presence of deletion can potentially induce a transition from a power law to an exponential regime. To account for both regimes, we assume p_k follows a power-law with an exponential correction, $p_k = Ck^{-\gamma}\Omega^k$. Next, we simplify the expression for the attachment kernels by setting $b, t = 1$, such that $\pi_k = A(a + k)$ and $f_k = B(t + k)$ and solve for γ and Ω in the limit $k >> 1$. To do so, we employ a high-degree expansion of the telescoping product p_k/p_{k-1} to leading orders in $1/k$ thus,

$$\begin{aligned} \frac{p_k}{p_{k-1}} &= \Omega\left(1 - \frac{\gamma}{k}\right) + O\left(\frac{1}{k^2}\right), \\ \frac{p_{k+1}}{p_{k-1}} &= \Omega^2\left(1 - \frac{2\gamma}{k}\right) + O\left(\frac{1}{k^2}\right). \end{aligned} \tag{S8}$$

Multiplying Eq.(3) by $1/p_{k-1}$, substituting this expansion and ignoring terms in $1/k$, yields the equation,

$$k(1-\Omega)(Ac+2mB-(r+2qm/\langle k\rangle)\Omega]+(Aca+B2mt)(1-\Omega)-Ac-B2mt \\ -\Omega+(r+2qm/\langle k\rangle)\Omega^2+\gamma[\Omega(Ac+2mB-2\Omega(r+2qm/\langle k\rangle)+(r+2qm/\langle k\rangle)]=0. \quad \text{(S9)}$$

Since (S9) must be true for all k, we can set the coefficient of k to zero, which gives two solutions for Ω, namely $\Omega=1$ and

$$\Omega=\frac{Ac+2mB}{r+2qm/\langle k\rangle}. \quad \text{(S10)}$$

If $\Omega<1$ then the solution (S10) is normalizable and p_k decays exponentially (with a power-law correction). However if the ratio is greater than 1, it does not correspond to a normalizable probability distribution and therefore the correct solution must be $\Omega=1$, leading to a purely power law distribution $p_k\sim k^{-\gamma}$.

To determine the expression for the power-law exponent γ, we set $\Omega=1$, the k-independent term in (S9) to zero, finding

$$\gamma=2+\frac{(1+r)[c(c+m(1-q))+a(c+m)(1+r)]}{c^2(1-r)+c[m(1-q)(3-r)-ar(1+r)]+m[2m(1-q)^2-a(1+r)(q+r)]}. \quad \text{(S11)}$$

S2.3 Stretched exponential at the critical point a_c

At the critical point a_c (Eq. (9)), p_k follows a stretched exponential. Making the ansatz,

$$p_k=Ck^{-\gamma}B^k\mathrm{e}^{-\zeta\sqrt{k}},$$

and once again employing the high-degree expansion, but this time expanding to order $k^{-3/2}$, we have

$$\frac{p_k}{p_{k-1}} = \Omega\left(1-\frac{\zeta}{2\sqrt{k}}-\frac{\gamma-\zeta^2/8}{k}-\frac{\gamma\zeta/2-\zeta/8-\zeta^3/48}{k^{3/2}}\right)+O\left(\frac{1}{k^2}\right),$$

$$\frac{p_k}{p_{k-1}} = \Omega\left(1-\frac{\zeta}{2\sqrt{k}}-\frac{\gamma-\zeta^2/8}{k}-\frac{\gamma\zeta/2-\zeta/8-\zeta^3/48}{k^{3/2}}\right)+O\left(\frac{1}{k^2}\right). \quad \text{(S12)}$$

As before we substitute this into Eq. (3) and find that for $\zeta\neq 0$, the terms of order k and $\sqrt{k}$ force $B=1$ and $r+2qm/\langle k\rangle=A(c+2m)$. Thus ζ can be non-zero only at the critical point. The term of order 1 gives,

$$\zeta=2\left(\frac{c+m(1-q)}{cr+m(q+r)}\right)^{1/2} \quad \text{(S13)}$$

and the term of order $k^{-1/2}$ gives

$$\gamma=-\frac{3}{4}+\frac{a}{2},$$

which reduces to the work of [27] in the special case $a=0, m=0$ and $r=1$.
